# Thymoquinone, a Novel Multi-Strike Inhibitor of Pro-Tumorigenic Breast Cancer (BC) Markers: CALR, NLRP3 Pathway and sPD-L1 in PBMCs of HR+ and TNBC Patients

**DOI:** 10.3390/ijms241814254

**Published:** 2023-09-19

**Authors:** Sawsan Elgohary, Reda A. Eissa, Hend M. El Tayebi

**Affiliations:** 1 Clinical Pharmacology and Pharmacogenomics Research Group, Department of Pharmacology and Toxicology, Faculty of Pharmacy and Biotechnology, German University in Cairo, Cairo 11835, Egypt; sawsanelgohary54@gmail.com; 2 Department of Surgery, Faculty of Medicine, Ain Shams University, Cairo 11591, Egypt; reissa75@gmail.com

**Keywords:** thymoquinone, PRR, calreticulin, NLRP3, PYCARD, capspase-1, IL-1β, sPD-L1, triple-negative breast cancer, hormone receptor-positive breast cancer

## Abstract

Breast cancer (BC) is not only a mass of malignant cells but also a systemic inflammatory disease. BC pro-tumorigenic inflammation has been shown to promote immune evasion and provoke BC progression. The NOD-like receptor (NLR) family pyrin domain-containing protein 3 (NLRP3) inflammasome is activated when pattern recognition receptors (PRRs) sense danger signals such as calreticulin (CALR) from damaged/dying cells, leading to the secretion of interleukin-1β (IL-1β). CALR is a novel BC biological marker, and its high levels are associated with advanced tumors. NLRP3 expression is strongly correlated with an elevated proliferative index Ki67, BC progression, metastasis, and recurrence in patients with hormone receptor-positive (HR+) and triple-negative BC (TNBC). Tumor-associated macrophages (TAMs) secrete high levels of IL-1β promoting endocrine resistance in HR+ BC. Recently, an immunosuppressive soluble form of programmed death ligand 1 (sPD-L1) has been identified as a novel prognostic biomarker in triple-negative breast cancer (TNBC) patients. Interestingly, IL-1β induces sPD-L1 release. BC Patients with elevated IL-1β and sPD-L1 levels show significantly short progression-free survival. For the first time, this study aims to investigate the inhibitory impact of thymoquinone (TQ) on CALR, the NLRP3 pathway and sPD-L1 in HR+ and TNBC. Blood samples were collected from 45 patients with BC. The effect of differing TQ concentrations for different durations on the expression of CALR, NLRP3 complex components and IL-1β as well as the protein levels of sPD-L1 and IL-1β were investigated in the peripheral blood mononuclear cells (PBMCs) and TAMs of TNBC and HR+ BC patients, respectively. The findings showed that TQ significantly downregulated the expression of CALR, NLRP3 components and IL-1β together with the protein levels of secreted IL-1β and sPD-L1. The current findings demonstrated novel immunomodulatory effects of TQ, highlighting its potential role not only as an excellent adjuvant but also as a possible immunotherapeutic agent in HR+ and TNBC patients.

## 1. Introduction

In 2020, breast cancer (BC) was ranked the most diagnosed cancer and the fifth-highest cause of cancer mortality worldwide [1]. Five molecular BC subtypes have been extensively characterized, comprising luminal A, with the best prognosis; luminal B/human epidermal growth factor receptor-negative (HER2-); luminal B/HER2+; HER-2 enriched [2]; and finally the most aggressive triple-negative breast cancer (TNBC) subtype, which lacks targeted therapy [2]. It has been shown that BC is not only an autonomous mass of epithelial cells but also a systemic inflammatory disease and a serious consequence of chronic inflammation [3,4,5]. Peripheral blood represents reservoirs and activation sites of immune cells during BC progression [6]. Interestingly, the protein expression profile of peripheral blood mononuclear cells (PBMCs) has been shown to be a reflection of its expression within the BC tissue itself [7]. Cancer cells perturb gene expression in PBMCs, leading to many systemic signaling and immune evasion issues in BC patients [8]. In tumor microenvironment, distinct inflammatory proteins and immune cells release various cytokines into the bloodstream, promoting systemic inflammation [9]. These chronic inflammatory actions may be triggered by danger signals called danger-associated molecular patterns (DAMPs) released from injured or dying cells, leading to the initiation of sterile inflammation and immunogenic cell death (ICD) [10,11]. TNBC is the most immunogenic subtype [12,13], while estrogen receptor + (ER+) BC, specifically luminal A, is the least immunogenic subtype [14]. Failure of DAMPs to generate an effective antitumor response might turn DAMPs into a tumor-promoting mechanism and enhance chronic inflammation [10,15]. DAMPs represent a large range of chemically unrelated mediators, such as calreticulin (CALR) and adenosine triphosphate (ATP) [16]. In healthy cells, CALR resides in the endoplasmic reticulum (ER), acting as a chaperone that corrects protein folding [17]. Various chemotherapeutics [18], radiation [19], and oncolytic peptides [20] induce CALR’s translocation to the cell surface, acting as a DAMP and producing an “eat-me signal” that stimulates the engulfment of apoptotic cells by phagocytes [21]. CALR is expressed in PBMCs, including activated peripheral blood T-cells [22], macrophages [23], immature DC, monocytes [24], plasma cells [25], and NK cells [26]. Interestingly, the exposed CALR has been shown to be subsequently released and detected in the extracellular milieu [27]. Recently, CALR has been addressed as a promising biological marker of BC and an indicator of BC staging and prognosis [28,29,30,31,32]. In addition, the literature reported that CALR expression was associated with more advanced tumors in a study of 228 BC samples [33]. Moreover, in a cohort of 33 patients with BC, high levels of CALR correlated with metastasis, especially in axillary lymph nodes [31], and mediated the invasive BC phenotype [34].

Inflammasomes are inflammatory signaling complexes made up of pattern recognition receptors (PRRs) that sense released DAMPs, leading to their oligomerization recruiting the apoptosis-associated speck-like protein containing a caspase recruitment domain (ASC) also called PYCARD (PYD and CARD domain-containing) and the effector caspase-1 [35], which then cleaves the pro-interleukin-1β (pro-IL-1β) into the mature active IL-1β [35]. The NOD-like receptor (NLR) family pyrin domain-containing protein 3 (NLRP3) is the most extensively studied inflammasome [35]. Numerous studies have demonstrated that CALR is an NLRP3 activator [36,37,38,39]. NLRP3 is expressed in PBMCs [40,41], including B-cells, T-cells, dendritic cells, monocytes (very weakly) [42,43], and macrophages [44]. NLRP3 hyperactivation in PBMCs is exhibited in various inflammatory diseases [45,46,47,48,49,50,51], distinct cardiovascular diseases [52,53,54,55], and enhanced systemic inflammatory states [52], and it was detected in PBMCs of postmortem coronavirus disease-19 (COVID-19) patients [56]. In addition, NLRP3 was found to be overexpressed in PBMCs of various cancer patients [57,58,59,60]. In BC, the clinical analysis showed that NLRP3 and PYCARD expressions were strongly associated with more aggressive clinicopathological factors such as tumor size and proliferative index Ki67 and contributed to BC progression, especially in luminal BC patients [61]. Furthermore, claudin-low BC patients displayed an elevated expression of NLRP3, which was correlated with poor survival [62]. In addition, NLRP3 activation promoted BC metastasis and contributed to immune system dysfunction [63,64]. 

IL-1β is one of the primary mediators of systemic inflammation [65]. Distinct studies detected its expression in PBMCs [65,66,67,68]. In patients with invasive BC, IL-1β production by peripheral blood cells was associated with lymphatic metastasis [69]. Enzyme-linked immunosorbent assay (ELISA) findings showed that IL-β expressions were significantly increased in the sera and plasma of BC patients compared to the control group [70,71] and were related to tumor size, clinical stage, histological grade, and lymph node metastasis [70]. In addition, patients with metastatic BC exhibited an increased production of IL-1β compared to patients with early BC [72]. Macrophages, particularly tumor-associated macrophages (TAMs), are one of the main cell types that secrete high levels of IL-1β [73,74], promoting endocrine- and chemo-resistance in ER+ BC [75,76,77] through significantly decreasing ERα levels [76]. 

Tumor evasion has been a critical characteristic of cancer progression and poor prognosis in TNBC [12,78,79]. The immune checkpoint programmed death-1 (PD-1), which is expressed on activated T-cells [80], acts as ”brakes” that protect against autoimmunity [81] via binding to its ligand, programmed death ligand 1 (PD-L1), subsequently delivering inhibitory signals to T-cells, which leads to its exhaustion and deactivation [82]. Surprisingly, tumor cells not only express PD-L1 on its surface, but also secrete a soluble form of PD-L1 (sPD-L1) with an immunosuppressive function [83] that can be generated by cleavage from the cell surface [84]. Lately, sPD-L1 has attracted much attention [85], and emerging evidence has addressed sPD-L1 as a marker of inflammation [86]. In 2021, findings manifested a significant correlation between tumoral PD-L1 and sPD-L1 in the serum of BC patients [87]. A recent study reported that sPD-L1 could be used as a noninvasive biomarker for evaluating the malignancy of TNBC [88] since its high serum levels were correlated with poor response to neoadjuvant chemotherapy in patients with TNBC [88]. The results of another study agreed with these findings, showing that high levels of sPD-L1 in peripheral blood were associated with poor prognosis in BC [87]. These outcomes were further confirmed in March 2021, when it was stated that plasma sPD-L1 levels were higher in recurrent/metastatic patients than those in early-stage patients [87]. Yongjing Chen et al. reported that sPD-L1 was detected in the supernatant of MDA-MB231 [84]. Interestingly, a study in 2023 showed that IL-1β induced sPD-L1 release and enhanced membrane PD-L1 levels [86]. BC patients with elevated IL-1β and sPDL-1 levels showed a significantly shorter progression-free survival [89]. 

Thymoquinone (TQ) is a natural pharmacologically active ingredient derived from *Nigella sativa* seeds [90]. It has been known for its chemo-preventive and antineoplastic effects in diverse types of cancer for more than 50 years, as reviewed by our research group [91]. In BC, TQ exerted cytotoxic effects against various BC cell lines [92,93,94,95,96,97] and showed selective cytotoxicity against BC cells compared to normal cells [98]. Another study confirmed these results, demonstrating that TQ exerted selective cytotoxicity against pancreatic cancer cell lines compared to the non-toxic effect against PBMCs even at 100 µM [99]. In addition, TQ showed proliferative effects and enhanced immunological properties in PBMCs and macrophages, respectively [99,100]. To date, no literature explored the impact of TQ on CALR expression in any study model or ailment. Recently, few studies investigated the inhibitory effect of TQ on the NLRP3 pathway in distinct in vivo and in vitro models [101,102,103,104,105,106]. In addition, a recent study showed that TQ significantly inhibited the protein expression of PD-L1 in the TNBC cell line [107]. In contrast, TQ’s inhibitory effects on inflammasome and sPD-L1 are not explored yet in BC. This study is the first to explore whether TQ could target the aforementioned pro-tumorigenic BC markers, which consist of the CALR, NLRP3 pathway with a focus on the downstream IL-1β expression and protein release in both PBMCs and TAMs isolated from TNBC and HR+ BC patients, respectively. The second purpose was to compare the protein levels of sPD-L1 in HR+ and TNBC patients. Finally, the inhibitory effect of TQ on sPD-L1 was further investigated. Overall, this study was performed using different TQ concentrations for distinct durations.

## 2. Results

### 2.1. TQ-Inhibited CALR in PBMCs of HR+ BC Patients

**Figure 1:**
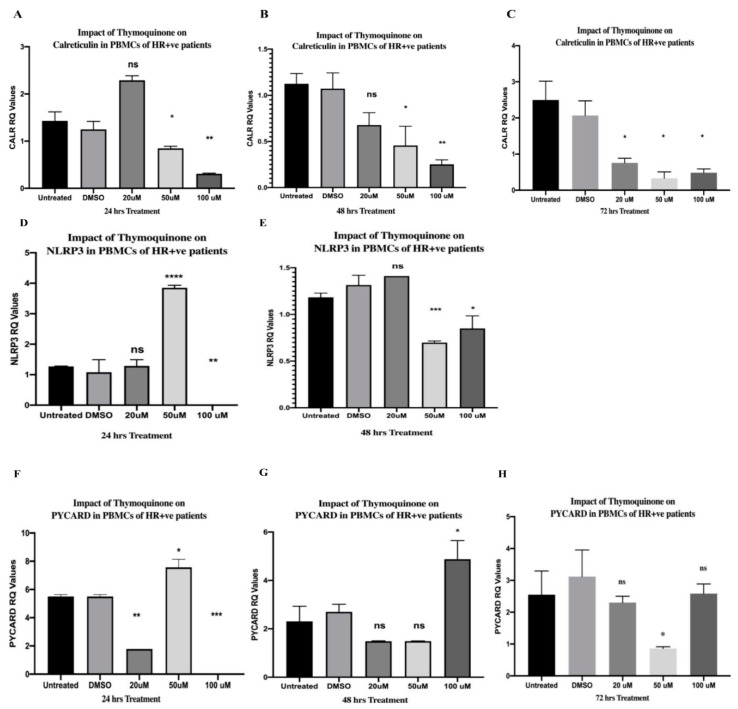
Inhibitory impact of TQ on CALR, NLRP3 and PYCARD expression in PBMCs of HR+ BC patients. (**A**–**C**) CALR expression versus TQ concentrations. PBMCs isolated from HR+ BC patients were treated with TQ (0, 20, 50, and 100 µM) for (**A**) 24 h, (**B**) 48 h, and (**C**) 72 h. (**D**–**E**) NLRP3 expression versus TQ concentrations. PBMCs isolated from HR+ BC patients were treated with TQ (0, 20, 50, and 100 µM) for (**D**) 24 h and (**E**) 48 h. (**F**–**H**) PYCARD expression versus TQ concentrations. PBMCs isolated from HR+ BC patients were treated with TQ (0, 20, 50, and 100 µM) for (**F**) 24 h, (**G**) 48 h, and (**H**) 72 h. All experiments were performed in triplicate, and data are expressed as the mean ± standard deviation. Statistical analysis was performed using the GraphPad Prism software version 9.1.1. The data were analyzed using one-way ANOVA and Dunnett’s multiple comparisons to compare each treated column and the DMSO control column (**** *p* value < 0.0001; *** *p* value < 0.001; ** *p* value < 0.01; * *p <* 0.05). TQ: thymoquinone; CALR: calreticulin; NLRP3: NOD-like receptor (NLR) family pyrin domain-containing protein 3 (NLRP3); PYCARD: PYD and CARD domain containing; PBMCs: peripheral blood mononuclear cells; HR+: hormone receptor-positive; BC: breast cancer; DMSO: dimethyl sulfoxide; ns: non-significant.

PBMCs isolated from HR+ BC patients were treated with increasing concentrations of TQ (20, 50, and 100 µM) for 24, 48, and 72 h. CALR expression was investigated compared to that of the DMSO control, and each concentration was performed in triplicate. After 24 h of TQ treatment, the 20 µM concentration resulted in a non-significant elevation in CALR expression. In contrast, the 50 µM (* *p* = 0.0195) and 100 µM (** *p* = 0.0013) concentrations significantly downregulated its expression (one-way analysis of variance (ANOVA), **** *p* < 0.0001; Figure 1A). After 48 h, the results showed a dose-dependent inhibition, which was significant at 50 µM (* *p* = 0.0345) and 100 µM (** *p* = 0.0043) (one-way ANOVA, ** *p* = 0.0033; Figure 1B). TQ significantly downregulated the expression of CALR at 20 µM (* *p* = 0.0414), 50 µM (* *p* = 0.0184), and 100 µM (* *p* = 0.0154) concentrations after 72 h of treatment (one-way ANOVA, ** *p* = 0.0045; Figure 1C).

### 2.2. TQ Significantly Downregulated CALR Expression in PBMCs Isolated from TNBC Patients in a Dose-Dependent Manner 

**Figure 2:**
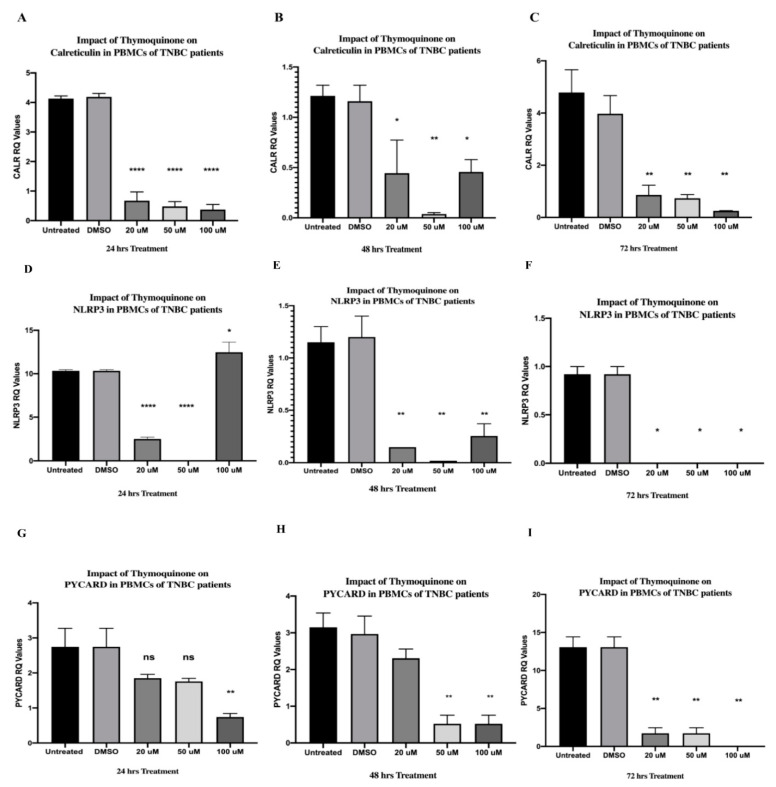
TQ significantly inhibited the expression of CALR, NLRP3 and PYCARD in PBMCs of TNBC patients. (**A**–**C**) CALR expression versus TQ concentrations. PBMCs isolated from TNBC patients were treated with TQ (0, 20, 50, and 100 µM) for (**A**) 24 h, (**B**) 48 h, and (**C**) 72 h. (**D**–**F**) NLRP3 expression versus TQ concentrations. PBMCs isolated from TNBC patients were treated with TQ (0, 20, 50, and 100 µM) for (**D**) 24 h, (**E**) 48 h, and (**F**) 72 h. (**G**–**I**) PYCARD expression versus TQ concentrations. PBMCs isolated from TNBC patients were treated with TQ (0, 20, 50, and 100 µM) for (**G**) 24 h, (**H**) 48 h, and (**I**) 72 h. All experiments were performed in triplicate and data were expressed as the mean ± standard deviation. Statistical analysis was performed using the GraphPad Prism software version 9.1.1. The data were analyzed using one-way ANOVA and Dunnett’s multiple comparisons to compare each treated column and the DMSO control column (**** *p* value< 0.0001; ** *p* value < 0.01; * *p <* 0.05). TQ: thymoquinone; CALR: calreticulin; NLRP3: NOD-like receptor (NLR) family pyrin domain-containing protein 3 (NLRP3); PYCARD: PYD and CARD domain containing; PBMCs: peripheral blood mononuclear cells; TNBC: triple-negative breast cancer; BC: breast cancer; DMSO: dimethyl sulfoxide; ns: non-significant.

TQ showed strong significant inhibition on the CALR expression after 24 h of treatment at 20 µM (**** *p* < 0.0001), 50 µM (**** *p* < 0.0001), and 100 µM (**** *p* < 0.0001) in a dose-dependent manner (one-way ANOVA, **** *p* < 0.0001) (Figure 2A). After 48 h of treatment, TQ significantly inhibited the expression of CALR at 20 µM (* *p* = 0.0357), 50 µM (** *p* = 0.0041), and 100 µM (* *p* = 0.0229) (one-way ANOVA, ** *p* = 0.0054) (Figure 2B). TQ showed a significant inhibition in the CALR expression after 72 h at 20 µM (** *p* = 0.0065), 50 µM (** *p* = 0.0033), and 100 µM (** *p* = 0.0029) in a dose-dependent manner (one-way ANOVA, ** *p* = 0.0016) (Figure 2C). The expression levels were compared to those of the DMSO control, and each TQ concentration was performed in triplicate. 

### 2.3. TQ Showed an Interesting Inhibitory Pattern in NLRP3 and PYCARD in PBMCs of HR+ BC Patients after 24 h of Treatment

The NLRP3 and PYCARD expressions were investigated with increasing TQ concentrations (20 µM, 50 µM, and 100 µM) after 24 h of treatment compared to those of the DMSO control, and each concentration was performed in triplicate. TQ caused a non-significant impact on NLRP3 expression at 20 µM; then, a significant increase was noticed at 50 µM (**** *p* < 0.0001), followed by a complete abolishment in its expression at 100 µM (** *p* = 0.0063) (one-way ANOVA, **** *p* < 0.0001) (Figure 1D). TQ showed a similar inhibitory pattern in PYCARD expression after 24 h of treatment (Figure 1F). At first, it was significantly downregulated at 20 µM (** *p* = 0.0059), followed by a significant elevation at 50 µM (* *p* = 0.0237). Finally, its expression was significantly abolished below the detection level at 100 µM (*** *p* = 0.0002)—one-way ANOVA of PYCARD expression after 24 h of treatment: **** *p* < 0.0001.

### 2.4. TQ Inhibited NLRP3 and PYCARD Expressions in PBMCs of HR+ BC Patients after 48 and 72 h

TQ caused an initial non-significant increase in NLRP3 expression at 20 µM, followed by a significant downregulation at 50 µM (*** *p* = 0.0006) and 100 µM (* *p* = 0.0266) after 48 h of treatment (one-way ANOVA, *** *p* = 0.0006) (Figure 1E). As for PYCARD, it showed a non-significant downregulation at 20 and 50 µM, followed by a significant increase at 100 µM (* *p* = 0.0172) after 48 h (one-way ANOVA, ** *p* = 0.0010) (Figure 1G). After 72 h of treatment, TQ caused a non-significant impact on PYCARD expression at 20 and 100 µM, whereas 50 µM significantly downregulated its expression (* *p* = 0.0245) (one-way ANOVA, *p* = 0.0389) (Figure 1H). The expression levels were compared to those of the DMSO control and each concentration was performed in triplicate.

### 2.5. TQ Strongly Inhibited NLRP3 Expression in PBMCs of TNBC Patients after 24 and 48 h of Treatment In Vitro and Completely Abolished Its Expression after 72 h 

TQ showed a strongly significant inhibition in NLRP3 at 20 µM (**** *p* < 0.0001) followed by a completely abolished expression at 50 µM (**** *p* < 0.0001). Unexpectedly, TQ suddenly upregulated the NLRP3 expression at 100 µM (* *p* = 0.0161) after 24 h of treatment (one-way ANOVA **** *p* < 0.0001) (Figure 2D). After 48 h of TQ treatment, NLRP3 was significantly inhibited at 20 µM (** *p* = 0.0050), 50 µM (** *p* = 0.0027), and 100 µM (** *p* = 0.0055) (one-way ANOVA, ** *p* = 0.0013) (Figure 2E). The NLRP3 expression was completely inhibited after 72 h of TQ treatments at 20 µM (*p = 0.0500), 50 µM (*p = 0.0500), and 100 µM (*p = 0.0500) (one-way ANOVA, * *p* = 0.0340) (Figure 2F). Each TQ concentration was performed in triplicate and the expression levels were compared to those of the DMSO control.

### 2.6. TQ Significantly Inhibited PYCARD Expression in PBMCs Isolated from TNBC Patients after 24 and 48 h of Treatment

After 24 h of TQ treatment, the 20 µM and 50 µM concentrations resulted in a non-significant difference in PYCARD expression. In contrast, the 100 µM concentration significantly downregulated its expression (** *p* = 0.0070) (one-way ANOVA, * *p* = 0.0108) (Figure 2G). PBMCs were further incubated for 48 h and showed a significant inhibition in PYCARD expression at 50 µM (** *p* = 0.0068) and 100 µM (** *p* = 0.0068), while the 20 µM concentration caused a non-significant inhibition (one-way ANOVA, ** *p* = 0.0044) (Figure 2H). TQ significantly inhibited the expression of PYCARD at 20 µM (** *p* = 0.0052) and 50 µM (** *p* = 0.0052) and completely abolished its expression after 72 h at 100 µM (** *p* = 0.0065) (one-way ANOVA, ** *p* = 0.0027) (Figure 2I). TQ concentrations were performed in triplicate and the expression levels were compared to those of the DMSO control.

### 2.7. TQ Significantly Inhibited Caspase-1 after 24, 48, and 72 h of Treatment in PBMCs of HR+ BC Patients

**Figure 3:**
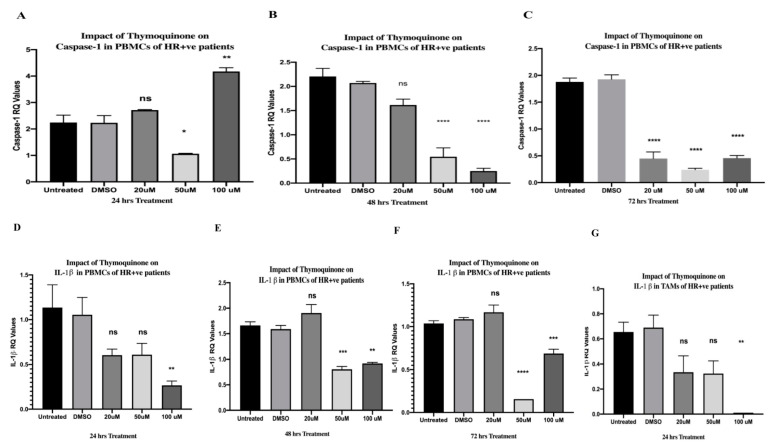
Inhibitory impact of TQ on caspase-1 and IL-1β expression in PBMCs and TAMs of HR+ BC patients, respectively. (**A**–**C**) Caspase-1 expression versus TQ concentrations. PBMCs isolated from HR+ BC patients were treated with TQ (0, 20, 50, and 100 µM) for (**A**) 24 h, (**B**) 48 h, and (**C**) 72 h. (**D**–**F**) IL-1β expression versus TQ concentrations. PBMCs isolated from HR+ BC patients were treated with TQ (0, 20, 50, and 100 µM) for (**D**) 24 h, (**E**) 48 h, and (**F**) 72 h. (**G**) Impact of TQ on IL-1β expression in TAMS isolated from HR+ BC patients after 24 h. All experiments were performed in triplicate and data are expressed as the mean ± standard deviation. Statistical analysis was performed using the GraphPad Prism software version 9.1.1. The data were analyzed using one-way ANOVA and Dunnett’s multiple comparisons to compare each treated column and the DMSO control column (**** *p* value< 0.0001; *** *p* value < 0.001; ** *p* value < 0.01; * *p <* 0.05). TQ: thymoquinone; HR+: hormone receptor-positive; BC: breast cancer; ns: non-significant; IL-1β: Interleukin-1 beta; PBMCs: peripheral blood mononuclear cells; TAMs: tumor-associated macrophages.

After 24 h of treating PBMCs isolated from HR+ BC patients with 20 µM TQ, an initial non-significant upregulation was observed in caspase-1, followed by a significant dampening in its expression at 50 µM (* *p* = 0.0195); finally, it was significantly upregulated at 100 µM (** *p* = 0.0013) (one-way ANOVA, *** *p* = 0.0002) (Figure 3A). After 48 h, TQ significantly inhibited caspase-1 expression in a dose-dependent manner with increasing TQ concentrations (50 and 100 µM) (**** *p* < 0.0001), whereas 20 µM caused a non-significant downregulation (one-way ANOVA, **** *p* < 0.0001) (Figure 3B). After 72 h of treatment, TQ showed a strongly significant downregulation of caspase-1 expression at 20, 50, and 100 µM (**** *p* < 0.0001) (one-way ANOVA, **** *p*< 0.0001) (Figure 3C). The expression levels were compared to those of the DMSO control and each concentration was performed in triplicate.

### 2.8. TQ Showed a Dose-Dependent Inhibitory Effect on Caspase-1 Expression in PBMCs Isolated from TNBC In Vitro 

**Figure 4:**
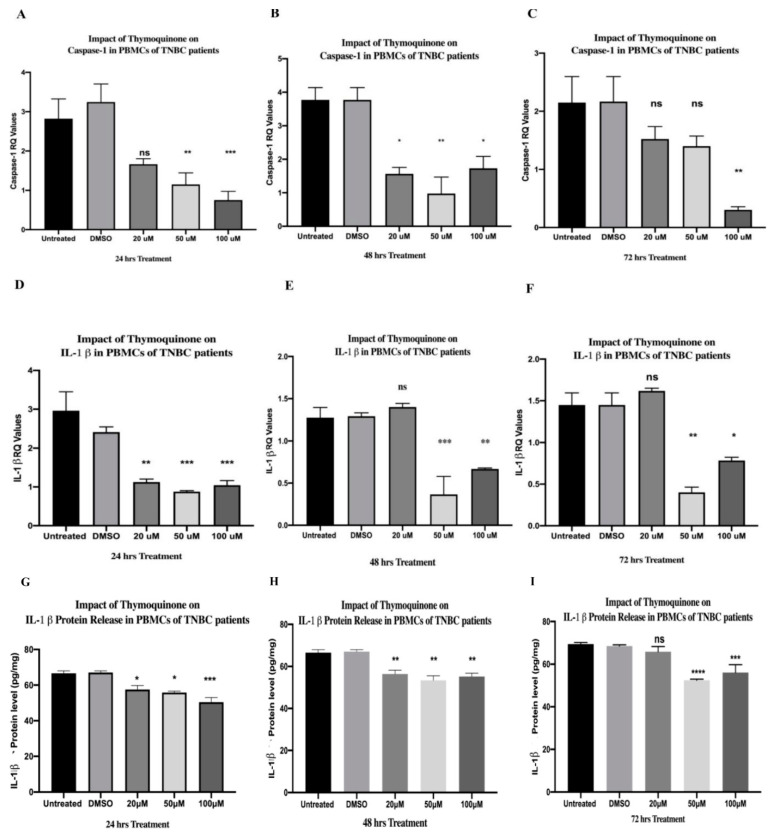
TQ inhibited caspase-1 expression, IL-1β expression, and protein release in PBMCs of TNBC patients. (**A**–**C**) Caspase-1 expression versus TQ concentrations. PBMCs isolated from TNBC patients were treated with TQ (0, 20, 50, and 100 µM) for (**A**) 24 h, (**B**) 48 h, and (**C**) 72 h. (**D**–**F**) IL-1β expression versus TQ concentrations. PBMCs isolated from TNBC patients were treated with TQ (0, 20, 50, and 100 µM) for (**D**) 24 h, (**E**) 48 h, and (**F**) 72 h. (**G**–**I**) IL-1β protein levels (pg/mL) secreted from PBMCs of TNBC patients treated with TQ concentrations (0, 20, 50, and 100 µM) for (**G**) 24 h, (**H**) 48 h, and (**I**) 72 h. All experiments were performed in triplicate, and data were expressed as the mean ± standard deviation. Statistical analysis was performed using the GraphPad Prism software version 9.1.1. The data were analyzed using one-way ANOVA and Dunnett’s multiple comparisons to compare each treated column and the DMSO control column (**** *p* value < 0.0001; *** *p* value < 0.001; ** *p* value < 0.01; * *p <* 0.05). TQ: thymoquinone; TNBC: triple-negative breast cancer; BC: breast cancer; ns: non-significant; IL-1β: Interleukin-1 beta; PBMCs: peripheral blood mononuclear cells; DMSO: dimethyl sulfoxide.

TQ showed a non-significant downregulation in caspase-1 expression at 20 µM, while TQ significantly dampened its expression at 50 µM (** *p* = 0.0065) and 100 µM (*** *p* = 0.0006) TQ concentrations after 24 h of treatment in vitro (one-way ANOVA, *** *p*= 0.0003) (Figure 4A). After 48 h of TQ treatments, caspase-1 expressions were significantly inhibited at 20 µM (* *p* = 0.0164), 50 µM (** *p* = 0.0015), and 100 µM (* *p* = 0.0254) (one-way ANOVA, *** *p* = 0.0010) (Figure 4B). PBMCs were further incubated for 72 h with the aforementioned TQ concentrations. Results showed non-significant differences in caspase-1 expression at 20 µM and 50 µM, whereas the 100 µM concentration significantly inhibited its expression (** *p* = 0.0013) (one-way ANOVA, ** *p* = 0.0014) (Figure 4C). TQ concentrations were performed in triplicate and the expression levels were compared to those of the DMSO control.

### 2.9. TQ Significantly Downregulated IL-1β Expression in a Dose-Dependent Manner in PBMCs of HR+ BC Patients 

After 24 h, TQ caused a non-significant difference at 20 µM or 50 µM. Interestingly, TQ significantly inhibited IL-1β at 100 µM (** *p* = 0.0012) (one-way ANOVA, ** *p* = 0.0011) (Figure 3D). Since TQ showed an inhibitory effect on IL-1β, the current study investigated longer treatment durations. Similarly, TQ significantly downregulated the expression of IL-1β at 50 µM and 100 µM after 48 h (*** *p* = 0.0008 and ** *p* = 0.0017, respectively). TQ strongly inhibited the expression of IL-1β after 72 h at 50 µM (**** *p* < 0.0001) and 100 µM (*** *p* = 0.0002), whereas the 20 µM concentration caused a non-significant change—one-way ANOVA of IL-1β expression after 48 and 72 h of treatment, **** *p* < 0.0001 (Figure 3E,F). The expression levels were compared to those of the DMSO control and each concentration was performed in triplicate.

### 2.10. TQ Significantly Downregulated IL-1β Expression in PBMCs of TNBCs 

TQ significantly downregulated the expression of IL-1β at 20 µM (** *p* = 0.0020), 50 µM (*** *p* = 0.0010), and 100 µM (*** *p* = 0.0007) after 24 h of treatment (one-way ANOVA, *** *p* = 0.0007) (Figure 4D). PBMCs were further incubated for 48 and 72 h. Results showed a non-significant upregulation in IL-1β at 20 µM after 48 h, while the 50 µM (*** *p* = 0.0005) and 100 µM (** *p* = 0.0071) concentrations significantly inhibited its expression (one-way ANOVA, *** *p* = 0.0001) (Figure 4E). TQ treatment for 72 h showed similar findings; there was a non-significant upregulation in IL-1β at 20 µM, while the 50 µM (** *p* = 0.0013) and 100 µM (* *p* = 0.0123) concentrations significantly inhibited its expression (one-way ANOVA of IL-1β, *** *p* = 0.0007) (Figure 4F). TQ concentrations were performed in triplicate and the expression levels were compared to those of the DMSO control. 

### 2.11. Microscopic and Flow Cytometry Results of Efficient CD14+ Monocyte Differentiation to Tumor-Associated Macrophages (TAMs) 

**Figure 5:**
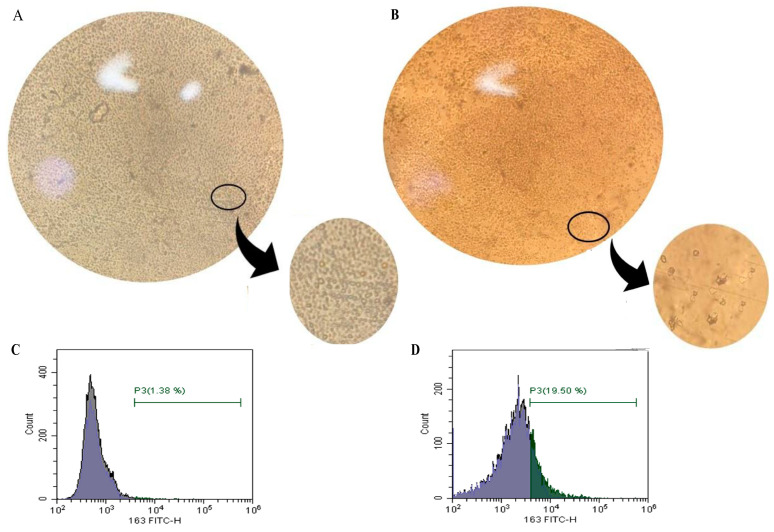
Efficiency of CD14+ monocyte differentiation to tumor-associated macrophages (TAMs). (**A**,**B**) Microscopic examination. (**A**) The figure shows freshly isolated and undifferentiated CD14+ monocytes characterized by relatively small size, with a spherical and smooth surface. (**B**) Differentiated TAMs morphology was observed on day 7 after culturing monocytes with culture media, tumor-conditioned media, IL-10, IL-4, and MCSF. Examination showed morphological changes; cells became relatively larger in size with edgy and rough surfaces confirming TAMs differentiation. (**C**,**D**) Flow cytometric analysis utilizing anti-CD163 FITC. (**C**) Freshly isolated undifferentiated CD14+ monocytes showed low CD163 positivity (1.38%). (**D**) After seven days of culturing monocytes with culture media, tumor-conditioned media, IL-10, IL-4, and MCSF, flow cytometry showed a 14-fold increase in CD163 positivity (19.50%) compared to that of undifferentiated CD14+ monocytes confirming efficient TAMs differentiation. TAMs: tumor-associated macrophages; BC: breast cancer; IL-10: interleukin-10; IL-4: interleukin-4; MCSF: macrophage-colony stimulating factor.

To validate that the cultured CD14+ monocytes were successfully differentiated to TAMs, microscopic examination and flow cytometry (CD163 positivity) were performed to compare monocytes to TAMs. Microscopic examination revealed morphological changes. The freshly isolated monocytes showed cells that were small in size with a spherical and smooth surface (Figure 5A). On day 7, cells showed TAMs morphology, which was larger in size relative to that of monocytes and had edgy and rough surfaces, showing efficient TAMs differentiation (Figure 5B).

To further confirm efficient TAMs differentiation and since CD163 is a TAMs biomarker [108], flow cytometry was performed to test the increase in CD163 positivity in TAMs relative to that of undifferentiated monocytes. Upon staining with anti-CD163, results showed that TAMs CD163 positivity increased and was even 14-fold higher than that of freshly isolated monocytes (19.5% CD163-positive versus 1.38%, respectively). Thus, TAMs morphology and increased CD163 positivity confirmed successful TAMs differentiation (Figure 5C,D).

### 2.12. TQ Significantly Abolished the Expression of IL-1β in TAMs Isolated from HR+ BC Patients 

TAMs are a major source of high IL-1β secretion [73,74], which causes a significant decrease in ERα levels [76], endocrine- and chemo-resistance in HR+ BC [75,76,77]. The impact of TQ on the expression of IL-1β, specifically in TAMs, was further investigated. TQ significantly abolished the expression of IL-1β in TAMs after treatment for 24 h at 100 µM (** *p* = 0.0014)—one-way ANOVA, ** *p* = 0.0018 (Figure 3G). The expression levels were compared to those of the DMSO control and each concentration was performed in triplicate.

Using ELISA, the current study further investigated the TQ’s impact on IL-1β protein release in PBMCs of HR+ BC patients and TAMs. 

### 2.13. TQ Significantly Downregulated Protein Release of IL-1β in PBMCs and TAMs of HR+ BC Patients 

**Figure 6:**
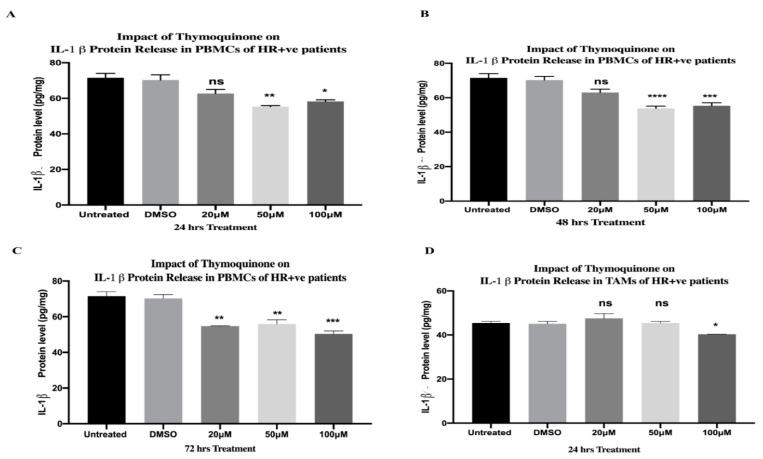
TQ significantly inhibited the protein release of IL-1β in PBMCs and TAMs of HR+ BC, respectively. (**A**–**C**) IL-1β protein level (pg/mL) released from PBMCs of HR+ BC patients treated with TQ concentrations (0, 20, 50, and 100 µM) for (**A**) 24 h, (**B**) 48 h, and (**C**) 72 h. (**D**) IL-1β protein level (pg/mL) released from TAMs of HR+ BC patients treated with TQ concentrations (0, 20, 50, and 100 µM) for 24 h. All experiments were performed in triplicate and data were expressed as the mean ± standard deviation. Statistical analysis was performed using the GraphPad Prism software version 9.1.1. The data were analyzed using one-way ANOVA and Dunnett’s multiple comparisons to compare each treated column and the DMSO control column (**** *p* value < 0.0001; *** *p* value < 0.001; ** *p* value < 0.01; * *p <* 0.05). TQ: thymoquinone; ns: non-significant; DMSO: dimethyl sulfoxide; PBMCs: peripheral blood mononuclear cells; TAMs: tumor-associated macrophages, HR+: hormone receptor-positive; BC: breast cancer; IL-1β: interleukin-1 beta.

After 24 h of TQ treatment, the 20 µM concentration caused a non-significant downregulation in the protein release of IL-1β, followed by a significant decrease at 50 µM (** *p* = 0.0025) and 100 µM (* *p* = 0.0102)—one-way ANOVA, *** *p* = 0.0009 (Figure 6A). TQ caused a non-significant downregulation, followed by a significant decrease at 50 µM (**** *p* < 0.0001) and 100 µM (*** *p* = 0.0008) after 48 h of treatment (one-way ANOVA, **** *p* < 0.0001) (Figure 6B). After 72 h of treatment, TQ significantly lessened the protein release of IL-1β at 20 µM (** *p* = 0.0046), 50 µM (** *p* = 0.0035), and 100 µM (*** *p* = 0.0008)—one-way ANOVA, *** *p* = 0.0002 (Figure 6C). The impact of TQ on the protein release of IL-1β was further investigated in TAMs of HR+ BC patients. A concentration of 20 and 50 µM of TQ showed a non-significant impact on IL-1β release. However, the 100 µM concentration significantly downregulated IL-1β protein release (* *p* = 0.0390)—one-way ANOVA, ** *p* = 0.0080 (Figure 6D). The protein levels were compared to those of the DMSO control, and each TQ concentration was performed in triplicate.

### 2.14. TQ Significantly Downregulated IL-1β Protein Release from PBMCs of TNBC Patients

TQ significantly downregulated the protein release of IL-1β after 24 h at 20 µM (* *p* = 0.0296), 50 µM (* *p* = 0.0134), and 100 µM (*** *p* = 0.008)—one-way ANOVA, ** *p* = 0.0013 (Figure 4G). Similarly, 20 µM (** *p* = 0.0067), 50 µM (** *p* = 0.0015), and 100 µM (** *p* = 0.0012) of TQ treatments significantly downregulated its protein release after 48 h (one-way ANOVA, *** *p* = 0.0007) (Figure 4H). After 72 h, 20 µM TQ did not cause a significant change in IL-1β protein release, while the 50 µM (**** *p* < 0.0001) and 100 µM (*** *p* = 0.005) concentrations significantly downregulated its release (one-way ANOVA, (**** *p* < 0.0001). (Figure 4I). TQ concentrations were performed in triplicate and the protein levels were compared to those of the DMSO control.

### 2.15. PBMCs of TNBC Patients Released Significantly Higher sPD-L1 Than That of HR+ BC Patients

**Figure 7:**
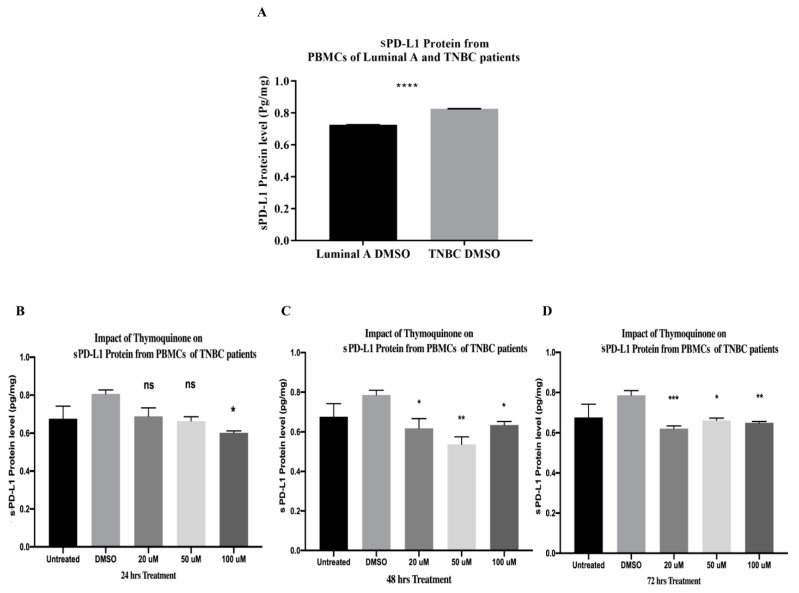
(**A**) sPD-L1 release in HR+ luminal A and TNBC patients. (**A**) sPD-L1 protein level (pg/mL) released from PBMCs of HR+ BC luminal A and TNBC patients in DMSO control. sPD-L1 from PBMCs of TNBC patients was significantly higher than that of luminal A HR+ BC patients (**** *p* < 0.0001). Data were analyzed using unpaired *t*-test. (**B**–**D**) TQ significantly inhibited sPD-L1 release from PBMCs of TNBC patients. ELISA quantified sPD-L1 protein level (pg/mL) released from PBMCs of TNBC patients treated with TQ concentrations (0, 20, 50, and 100 µM) for (**B**) 24 h, (**C**) 48 h, and (**D**) 72 h. All experiments were performed in triplicate and data were expressed as the mean ± standard deviation. Statistical analysis was performed using the GraphPad Prism software version 9.1.1. The data were analyzed using one-way ANOVA and Dunnett’s multiple comparisons to compare each treated column and the DMSO control column (**** *p* value < 0.0001; *** *p* value < 0.001; ** *p* value < 0.01; * *p <* 0.05). TQ: thymoquinone, HR+: Hormone receptor-positive; TNBC: triple-negative breast cancer; BC: breast cancer; ns: non-significant; sPD-L1: soluble programmed death ligand 1; PBMCs: peripheral blood mononuclear cells; DMSO: dimethyl sulfoxide; ELISA: enzyme-linked immunosorbent assay.

The current study compared the release of sPD-L1 from PBMCs of TNBC patients in DMSO control and that of luminal A HR+ via ELISA. Results showed that PBMCs of TNBC patients released significantly higher protein levels of sPD-L1 than those of luminal A HR+ BC patients (**** *p* < 0.0001). Data were analyzed using an unpaired *t*-test (Figure 7A). Thus, the present study investigated the impact of increasing TQ concentrations for various durations on sPD-L1 in TNBC only. 

### 2.16. TQ Significantly Downregulated sPD-L1 Release from PBMCs of TNBC Patients after 24, 48, and 72 h of Treatment In Vitro

After 24 h of treatment, TQ showed a non-significant impact on sPD-L1 from PBMCs of TNBC patients at 20 µM and 50 µM. However, 100 µM significantly downregulated its protein level (* *p* = 0.0468) (Figure 7B). After 48 h of TQ treatments, 20 µM (* *p* = 0.0435), 50 µM (** *p* = 0.0014), and 100 µM (* *p* = 0.0300) significantly downregulated sPD-L1 release (one-way ANOVA, *p* = 0.0066) (Figure 7C). After 72 h of treatment, TQ caused a significant downregulation in sPDL-1 protein release at 20 µM (*** *p* = 0.0008), 50 µM (* *p* = 0.0101), and 100 µM (** *p* = 0.0047)—one-way ANOVA, *p* = 0.0037 (Figure 7D). TQ concentrations were performed in triplicate and the protein levels were compared to those of the DMSO control.

## 3. Discussion

More than approximately 60% of the available anticancer drugs were derived from natural sources, in one way or another [109]. Plant-derived agents such as vincristine and paclitaxel are among the most effective cancer chemotherapeutics [110]. Interestingly, various chemotherapeutics led to the secretion of danger signals [111] such as CALR [16] which showed various tumor-promoting effects in BC and has been recently addressed as a biological marker of BC [28,29,30,31,32,33,34]. CALR has been reported to be an activator to the NLRP3 pathway [36,37,38,39]. In BC, NLRP3 and PYCARD expressions were strongly associated with pro-tumorigenic and aggressive clinicopathological features in luminal [61] and TNBC patients [62]. NLRP3 activation promoted immune system dysfunction [64], BC growth, enhanced angiogenesis [63], migration [112], and BC metastasis [63,64]. The downstream IL-1β was correlated with large tumor size, clinical stage, histological grade [70,72], endocrine- and chemo-resistance in HR+ BC patients [75,76,77]. Moreover, IL-1β production by peripheral blood cells enhanced lymphatic metastasis in BC [69]. Recently, sPD-L1 has attracted much attention [85] since its high serum levels were correlated with metastasis, immunosuppression, and poor prognosis in TNBC patients [87,88]. Interestingly, IL-1β induced sPD-L1 release [86]. Elevated IL-1β and sPDL-1 levels showed a significantly shorter progression-free survival in BC patients [89], highlighting the beneficial impacts of targeting IL-1β in both HR+ and TNBC patients. It has been shown that BC is a systemic inflammatory disease [3,4,5], where peripheral blood represents reservoirs and activation sites of immune cells during BC progression [6]. CALR [22,23,24,25,26], NLRP3 [45,46,47,48,49,50,51], and IL-1β [65,66,67,68] have been shown to be expressed in PBMCs and were addressed as markers of systemic inflammation [52,113,114], as well as sPD-L1 that was recently indicated as a sign of inflammation [86]. The current study aimed to target the aforesaid pro-tumorigenic BC markers comprising CALR, NLRP3 complex, sPD-L1, and IL-1β in PBMCs and TAMs of TNBC and HR+ BC patients, respectively. NLRP3 required two signals for activation: the first one was the priming step that needed the transcription of NLRP3 components and pro-IL-1β via NF-κB [115,116], while the second signal was through various danger signals that led to the oligomerization of inflammasome components and the formation of an NLRP3-active complex, and then, finally, the secretion of IL-1β [115,116,117]. For the first time, the present study explored the inhibitory impact of TQ on the aforementioned components since it suppressed NF-kB activation in BC [118] and dampened NLRP3 in human and mouse melanoma in vitro [101]. In addition, a recent study discussed the inhibitory impact of TQ on PD-L1 in the TNBC cell line [107].

**Figure 8:**
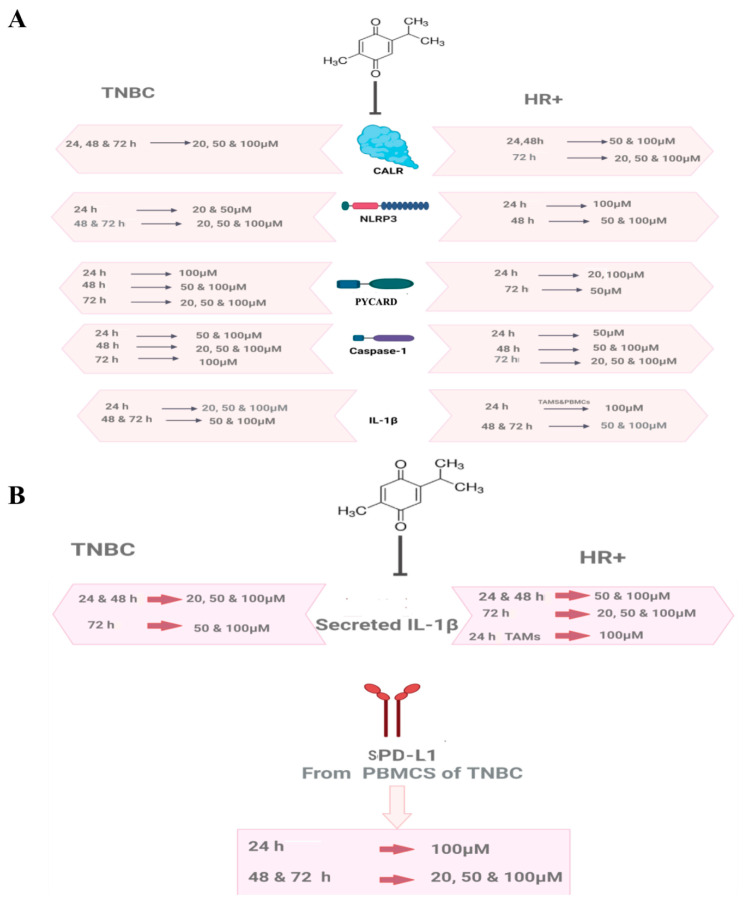
Summary of TQ’s significant inhibitory concentrations for distinct durations on CALR, inflammasome pathway, and sPD-L1 in HR+ and TNBC. (**A**) TQ’s significant inhibitory concentrations on expression of CALR, NLRP3 complex components and IL-1β in PBMCs/TAMs of HR+ and TNBC after 24, 48, and 72 h of treatment. (**B**) The significant inhibitory concentrations of TQ after 24, 48, and 72 h of treatment on IL-1β and sPD-L1 secretion from TAMs/PBMCs of HR+ and TNBC, respectively. The expression and protein levels were compared to those of the DMSO control. All experiments were performed in triplicate. TQ: thymoquinone; CALR: calreticulin; NLRP3: NOD-like receptor (NLR) family pyrin domain-containing protein 3 (NLRP3); PYCARD: PYD and CARD domain containing; PBMCs: peripheral blood mononuclear cells; TAMs: tumor-associated macrophages; HR+: hormone receptor-positive; TNBC: triple-negative breast cancer; BC: breast cancer; DMSO: dimethyl sulfoxide; IL-1β: interleukin-1 beta; sPD-L1: soluble programmed death ligand 1.

CALR resides in the ER, acting as a chaperone in healthy cells [17]. When cells are exposed to stress/injury such as chemotherapy [18], radiation [19], and oncolytic peptides [20], CALR translocates to the cell surface, acting as a DAMP [21,119]. The translocated CALR can be released into the extracellular milieu [27]. Recently, CALR has been addressed as a novel biological BC marker and an indicator of BC staging and prognosis [28,29,30,31,32]. Moreover, CALR expression was associated with more advanced BC tumors [33] and mediated invasive BC phenotype [34]. In addition, it was correlated with enhanced metastasis in BC patients [31]. Notably, ER contains other chaperones that assist in protein folding processes, such as glucose-regulated protein GRP78 [120]. Like in the case of CALR, stressful conditions upregulated GRP78’s translocation on the surface of the cell membrane [121], and it was elevated in endocrine-resistant BC that directly affected the responsiveness to anti-estrogen therapy [122]. In TNBC, GRP78 has been shown to interact with PD-L1 at the ER region and increase its levels [123]. The current study showed that TQ was a successful inhibitor of CALR expression in PBMCs of HR+ and TNBC patients in various concentrations and durations, as shown in Figure 8A. Although this is the first study to report the inhibitory effect of TQ on CALR in general and specifically in BC, the literature reported that TQ inhibited the expression of GRP78 in a rat model in vivo [124]. A study in multiple myeloma showed that high CALR expression was associated with an increased PD-L1 level [125]. Another study noticed that doxorubicin enhanced CALR and PD-L1 expression in BC cells in a dose-dependent manner [126], highlighting the potential role of CALR in immunosuppression and resistance. Interestingly, a recent study elaborated that tumors release a soluble CALR [127] that acts as a decoy for CALR receptors in phagocytes inhibiting the uptake of dying cancer cells leading to the accumulation of immunosuppressive cells in peripheral blood [128], immune evasion [129], and resistance to PD-1/PD-L1 blockade [127]. This raises a number of questions that require urgent investigation: Does soluble CALR interact with sPD-L1 aggravating immunosuppression? Does CALR interact with PD-L1 in the ER, causing immunotherapy resistance in BC? Also, it would be worth further examining the effect of TQ on CALR/PD-L1 and GRP78/PD-L1 expression in TNBC patients in vivo.

Various studies demonstrated that CALR is an NLRP3 activator [36,37,38,39]. The inhibitory impact of TQ on NLRP3 in BC was not investigated to date. It has been noticed that the most common concentrations of TQ in various BC cell lines ranged from 20 to 100 µM [118,130,131,132,133,134] and were commonly incubated for 24, 48, and 72 h [93,98,130,135,136,137]. In addition, a recent study showed that TQ was not cytotoxic to PBMCs at concentrations of 100, 50, 25, 12.5, 6.25, 3.125, 1, and 0.1 μM (medium containing ≤0.1% DMSO) incubated for 24, 48, and 72 h [99]. Interestingly, TQ has even been shown to proliferate PBMCs [99]. Another study stated that 50 and 100 µM TQ concentrations enhanced monocyte-derived macrophage activity and noticed an increase in the phagocytotic abilities after the TQ treatment [100]. Thus, 20, 50, and 100 µM TQ concentrations incubated for the aforementioned durations were chosen in this study. NLRP3 expression was significantly inhibited dose-dependently by TQ in PBMCs of HR+ BC patients and TNBC patients, as summarized in Figure 8A. The aforementioned results came in accordance with those for A375 and B16F10 melanoma cells [101], where 5–20 μM of TQ significantly decreased NLRP3 expression in a dose-dependent manner [101]. In view of the fact that TNBC and HR+ subtypes are immunohistochemically distinct [138,139,140,141,142], it has been noticed that after 24 h of TQ treatment, 50 µM TQ upregulated NLRP3 followed by a completely abolished expression at 100 µM in PBMCs of HR+ BC (Figure 1D). On the contrary, in PBMCs of TNBC, 50 µM abolished its expression, followed by significant upregulation at 100 µM after 24 h of TQ treatment (Figure 2D). This demonstrates that TQ’s inhibitory concentrations differ from one BC subtype to another. As for PYCARD expression, TQ significantly downregulated its expression in PBMCs of HR+ and TNBC patients (Figure 8A). This came in accordance with an in vivo study where TQ significantly downregulated the PYCARD expression in rats fed with ethanol and a high-fat diet [102]. Notably, 50 µM and 100 µM TQ incubated for 24 h (Figure 1F) showed a similar pattern to that of the NLRP3 expression in HR+ PBMCs (Figure 1D), where it significantly upregulated the PYCARD expression at 50 µM, followed by a completely abolished PYCARD expression at 100 µM (Figure 1F). 

Caspase-1 is an inflammatory caspase that is activated via inflammasome complexes in response to DAMPs or pathogen-associated molecular patterns (PAMPs) [143]. Since it is a protease, it cleaves the proinflammatory cytokine IL-1β into its active form after its activation [143]. TQ significantly inhibited caspase-1 expression in PBMCs of HR+ and TNBC (Figure 8A), which came in accordance with distinct in vivo and in vitro studies [101,103,104]. In BC, high levels of IL-1β showed various tumor-promoting impacts [144,145,146]. Peripheral blood cells of invasive BC patients produced high levels of IL-1β, leading to metastasis [69]. In sera and plasma of BC patients, ELISA findings showed that IL-β expressions were significantly increased compared to those in the control group [70,71]. Remarkably, IL-1β is secreted via not only the inflammasome pathway [143], but also NF-κB [115,116] which is activated via extracellular ATP [147] and the purinergic receptor P2X7R [148]. The released IL-1β was found to promote the production of pro-IL-1β by binding to the IL-1 receptor, which is expressed in various BC cells, including MDA-MB231 [149]. TAMs are a major source of IL-1β expression and secretion [73,74] and important BC promoters [150]. HR+ BC patients exhibited high levels of IL-1β leading to metastasis [77], downregulation of ERα [76], chemo-resistance, and endocrine resistance [77], which has been shown to be a great challenge in the treatment of hormonal BC patients [151]. In addition, IL-1β provoked progression in TNBC and its inhibition showed synergistic impacts in combination with a PD-L1 blocker [152]. Thus, in the current study, there was a focus on IL-1β in PBMCs and TAMs of TNBC and HR+ BC patients, respectively. Because IL-1β is secreted via various pathways, TQ as an inhibitor to NFκB [118] and NLRP3 in melanoma [101] was an excellent candidate to lessen its expression in the current study. Moreover, the literature demonstrated that patients with BC might experience a change in their BC subtype after neoadjuvant chemotherapy (nCT), leading to a change in adjuvant treatment in 100% of such patients [153]. That is why the HER2 and HR status, including the ER and the progesterone receptor (PR), should be evaluated not only before the initiation of nCT, but also after nCT [153]. Since IL-1β contributed to a significant decrease in ERα levels [76], further studies should be conducted to unravel the hidden reasons for such BC subtype conversion after nCT and clarify whether inflammasome pathway and the subsequent IL-1β secretion are responsible for such change. The current study showed that TQ significantly inhibited IL-1β expression and protein release in TAMs and PBMCs isolated from HR+ and TNBC patients, respectively (Figure 8A,B). The aforementioned inhibitory impacts on IL-1β came in accordance with distinct in vivo and in vitro studies [101,102,103,104,105,154].

To date, the current study is the only available investigation of TQ’s impact on NLRP3 in BC. However, various research articles examined its inhibitory effect on NLRP3 in various models [101,102,103,104,105,106]. One study examined its inhibitory impact on NLRP3 in human and mouse melanoma in vitro with increasing concentrations of TQ: 5, 10, and 20 µM for a fixed duration (24 h); TQ showed a dose-dependent inhibitory effect [101]. In the pancreatitis model, rats were treated with 100 mg/kg orally [102] and an oral TQ dose of 50 mg/kg/day in acute kidney injury [103] and cardiac damage in mice models in vivo [104,105]. In the Alzheimer’s rat model, oral administration of 10 mg/kg TQ ameliorated neuroinflammation via suppressing mRNA and protein levels of NLRP3 and IL-1β [106]. Interestingly, TQ significantly inhibited the expression of NLRP3 and its downstream proteins in all in vivo models [102,103,104,105,106]. The present study showed that TQ inhibited NLRP3 complex components in BC, which came in accordance with the aforementioned studies [101,102,103,104,105,106].

Along with the current study, various research papers showed that TQ is a possible multi-strike inhibitor of the NLRP3 pathway. It inhibited NFκB [118], which was pivotal in the priming step of NLRP3 activation [115,116]. Ben-Wen Cui et al. showed that TQ significantly suppressed the protein expression of IL-1β and the purinergic receptor P2X7R [155], which is a well-known activator of NLRP3 [156]. Various studies reported that the ATP-sensitive K+ channel is among the activators of NLRP3, where its inhibition by Glyberide [157] successfully suppressed NLRP3 activation [157,158]. However, the present study showed, for the first time, that TQ was a successful inhibitor to the danger signal CALR, NLRP3 and its downstream components. Nevertheless, Suddek et al. reported that TQ activated the K+ ATP channels [159]. These results came in accordance with those of another study reporting the participation of K+ ATP channels in the pharmacological effects of TQ [160]. The aforementioned impacts of TQ on the K+ ATP channel might explain the abrupt upregulation and the interesting inhibitory pattern of TQ in PBMCs of HR+ and TNBC subtypes (Figure 1D,F, Figure 2D and Figure 3A). In addition, it has been remarked that 20 µM of TQ showed a non-significant elevation in CALR expression after 24 h of treatment in PBMCs of HR+ BC patients (Figure 1A), highlighting that the TQ-induced K+ ATP channel activation [159] might be responsible for such upregulation. It was believed that mitochondrial and cytoplasmic ATP-dependent K+ channels were structurally close [161]. Shigaeva et al. reported that CALR participated in mitochondrial ATP-dependent K+ Channel transport, but the exact function and mechanism were unclear [161]. Since TQ activated ATP-sensitive K+ channel [159], it would be very interesting to examine the effect of TQ on K+ ATP channel/CALR and whether this channel was responsible for the non-significant upregulation of CALR after 24 h of treatment. 

Recently, it has been shown throughout various studies that high levels of sPD-L1 are associated with a poor prognosis in TNBC patients [87,88]. In March 2021, ELISA findings showed that sPD-L1 released from the TNBC subtype “MDA-MB231” was higher than that of luminal A T47D and MCF-7 BC cell lines [87]. The current study showed that sPD-L1 released from PBMCs of TNBCs BC patients was significantly higher than that of luminal A (**** *p* < 0.0001) (Figure 7A); these results came in accordance with those of Baojuan Han et al. [87]. For the first time, the present study investigated the impact of increasing TQ concentrations for various durations on protein levels of sPD-L1 released from PBMCs of TNBC patients via ELISA. Fortunately, TQ significantly downregulated sPD-L1 protein release in PBMCs of TNBC patients after 24, 48, and 72 h of treatment (Figure 8B). Although this is the first study that showed the inhibitory impact of TQ on sPD-L1 in PBMCs of TNBC patients, a recent study reported that TQ significantly reduced PD-L1 expression in MDA-MB231 cells [162], which came in accordance with the current results. Thus, collectively, these results suggest that TQ might be a novel adjuvant with atezolizumab (Tecentriq), a PD-L1 inhibitor, in TNBC. 

The literature showed that CALR increased PD-L1 levels [125]. In addition, doxorubicin enhanced the CALR and PD-L1 expression in BC cells [126]. In 2019 and 2020, FDA approved the use of atezolizumab (Tecentriq) [163] and pembrolizumab (Keytruda), a PD-1 inhibitor [164], in combination with chemotherapy in the treatment of metastatic PD-L1-positive TNBC patients [165]. Studies showed that IL-1β induced sPD-L1 release in patients with BC [86] and was correlated with significantly short progression-free survival [89]. In addition, NLRP3 contributed to immunosuppression [166] and promoted the expression of PD-L1 in various cancers including BC [59]. Moreover, tumor PD-L1, PD-1, or PD-L1 im-munotherapy blockade activated NLRP3 leading to resistance in BC as reviewed by our research group [167], highlighting the fact that CALR/NLRP3 and PD-L1/sPD-L1 might provoke an immunosuppressive/resistance loop in BC. The current findings manifested TQ as a multi-strike inhibitor of CALR, the NLRP3 pathway and sPD-L1 secretion in BC. 

The phosphoinositide 3-kinase (PI3K)/AKT pathway is a regulator of pivotal cell functions such as cell proliferation and survival [168]. In BC, PI3K/AKT deregulation via mutations in the PIK3CA gene or inactivation of the tumor suppressor phosphatase and tensin homolog deleted on chromosome 10 (PTEN) have been common in ER+ and TNBC patients, respectively [169,170]. It has been reported that an increased PD-L1 expression was mediated via PI3K/AKT activation or a knockdown of PTEN [171]. In addition, PD-L1 activated PI3K/AKT in colorectal cancer [172]. Distinct studies reported that PI3K/AKT increased the levels of matrix metalloproteases (MMP) [173,174]. Surprisingly, tumor cells solubilize the membrane-bound PD-L1 and secrete sPD-L1 [83] by cleavage from the cell surface by MMP [84]. A recent study reported that IL-1β induced sPD-L1 release and increased PD-L1 levels via MMP [86]. Another study came in accordance and showed that IL-1β enhanced MMP production [175,176], leading to PD-L1 solubilization and secretion [84]. In addition, NLRP3 directly activated MMP [177]. Recent findings reported that PI3K/AKT inhibition suppressed the activation of NLRP3 and decreased its expression [178]. In various cancer studies, TQ has been reported to be a PI3K/AKT inhibitor [179,180,181], including BC via PTEN upregulation [93,134]. Furthermore, TQ significantly inhibited MMP in numerous cancers such as BC [182], hepatocellular carcinoma [183], prostate cancer [184], renal cell carcinoma [185], neuroblastoma [186], lung cancer [187], and glioblastoma [188]. Recently, FDA approved the use of the oral PI3K inhibitor alpelisib (Piqray) in the treatment of HR+ metastatic BC patients with mutated PIK3CA [189], collectively providing a hint at the potential use of TQ in combination with alpelisib. Also, various in vitro studies should urgently proceed to examine the synergistic impact of combining TQ with alpelisib in distinct TNBC and HR+ BC patients. In addition, it would be worth examining the effect of PI3K/AKT on MMP/sPD-L1 in both HR+ and TNBC with or without TQ. Finally, it is interesting to investigate whether alpelisib would affect the levels of sPD-L1 in TNBC.

The chemokine receptor 2 (CXCR2) has been shown to promote therapy resistance and suppress immunotherapy [190]. The literature reported that CXCR2 activates NLRP3 [191]. Recently, a study performed by our research group showed that doxorubicin increased CXCR2 expression in MDA-MB-231 [190]. In addition, CXCR2 inhibition significantly improved the efficacy of atezolizumab in TNBC in vitro [190]. Another study showed that CXCR2 knockdown decreased PD-L1 levels [192]. Interestingly, 100 μM of TQ significantly suppressed CXCR2 mRNA levels [193]. Thus, the inhibitory effect of TQ on CXCR2/NLRP3/PD-L1 and sPD-L1 should be further investigated in BC. In addition, the impact of TQ on CALR/NLRP3/IL-1β/MMP and sPD-L1 needs closer consideration. Also, it would be worth investigating the impact of combining TQ/doxorubicin and atezolizumab in BC. 

Cyclin D is frequently deregulated in human cancer and promotes cell division by activating cyclin-dependent kinase 4/6 (CDK 4/6), causing enhanced cell proliferation and BC progression [194,195]. In clinical trials, novel CDK 4/6 inhibitors showed remarkable impacts and received FDA approval for treating BC, such as Ribociclib^®^, Palbociclib^®^, and Abemaciclib^®^ [194]. Studies showed that NLRP3 inhibition suppressed cyclin D1 [196]. In addition, TQ has been reported to induce cell cycle arrest through the inhibition of cyclin E, cyclin D, and cyclin-dependent kinase 2 (CDK-2) in various cancers, as reviewed by our research group [91]. Since the current study showed that TQ significantly inhibited NLRP3 in BC, it would be beneficial to investigate the impact of TQ/cyclin D on CDK4/6 in combination with Abemaciclib^®^ in BC, as it might provide a synergistic effect.

To date, no clinical trials have investigated the effect of TQ on cancer in general or BC patients in particular. However, according to clinical trials.gov, TQ has been evaluated for its clinical, immunohistochemical, and chemo-preventive impacts on potentially malignant oral lesions. However, the results are not published yet. Patients were divided into three equal groups: Group A was administered buccal tablets containing 10 mg TQ; Group B received buccal 5 mg TQ tablets; Group C was the placebo control group (ClinicalTrials.gov Identifier: NCT03208790).

Among the limitations of the present study is the fact that it only focused on targeting the pro-tumorigenic BC markers comprising CALR, NLRP3 complex components, IL-1β, and sPD-L1. However, the exact mechanism of how TQ significantly exerted its inhibitory action is still unclear. In addition, it is still questionable whether there is a correlation between PI3K/AKT, CALR/NLRP3/IL-1β, and MMP/sPD-L1/PD-L1 and cyclin D/CDK4,6 in PBMCs of BC patients. Since TQ significantly inhibited CALR and sPD-L1, it would be beneficial to examine its impact on soluble CALR, PD-L1 expression and protein expressions of CALR, NLRP3, PYCARD, and caspase-1. Moreover, it would be worth examining the inhibitory effect of TQ on NLRP3 expression in PBMCs of HR+ BC patients after 72 h of treatment and IL-1β in TAMS of TNBC patients. Further in vitro investigation should be preceded to choose TQ’s most potent concentration for the most suitable duration in BC. Also, its inhibitory impact should be explored in the HER2+ subtype. The current findings showed that TQ exerted its significant inhibition in PBMCs and TAMs of BC patients. Thus, investigation should be proceeded in BC tissues and in BC patients.

## 4. Materials and Methods

### 4.1. Sample Collection

A total number of 45 BC patients provided written informed consent. Patients diagnosed with TNBC and HR+ luminal A BC were included in the present study. The other BC subtypes were excluded. Patients were stratified into HR+ BC and TNBC patients. All patients were females between the ages of 30 and 79 years. Clinical features are presented in Table 1. Approximately 10 mL of fresh blood was collected from each patient in EDTA tubes to prevent blood coagulation. The current study was approved by the Ethical Committee of the German University in Cairo (approval no. PTX-2019-01-HET) and the Ain Shams University (Cairo, Egypt) and followed the ethical guidelines of the 1975 Declaration of Helsinki. 

### 4.2. Peripheral Blood Mononuclear Cell Isolation 

**Figure 9:**
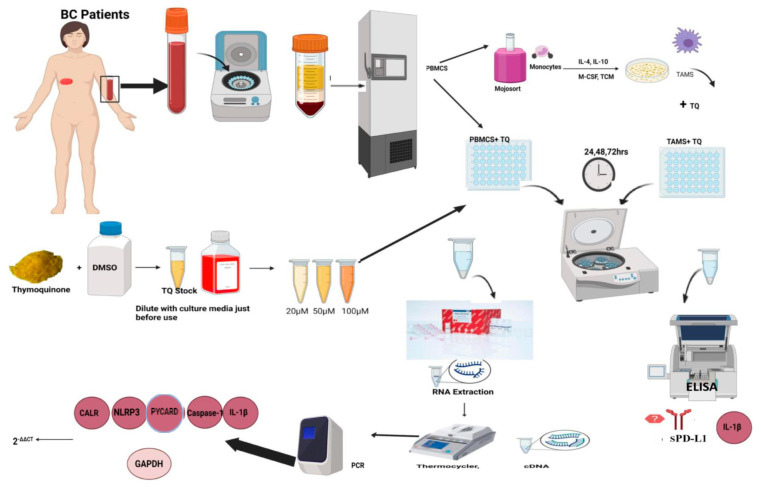
Simplified diagram of methodology. Blood samples were collected from 45 patients with breast cancer (BC): 30 were luminal A and 15 were triple-negative breast cancer patients (TNBC). Peripheral blood mononuclear cells (PBMCs) were isolated from blood and stored at −80 °C for future use. Monocytes were extracted from PBMCs using a MojoSort monocyte isolation kit and then differentiated into tumor-associated macrophages (TAMs). Thymoquinone (TQ) stock solution was prepared in dimethyl sulfoxide (DMSO) and was diluted with fresh culture media just before use. According to the literature, 20, 50, and 100 µM concentrations were prepared. PBMCs and TAMs were cultured with the aforementioned TQ concentrations for 24, 48, and 72 h. RNA isolation was conducted with RNeasy Mini Kit (QIAGEN) and then converted into cDNA in the thermocycler. Finally, RT-PCR was used to quantify calreticulin, NLRP3, PYCARD, caspase-1, and IL-1β. Relative Quantitation (RQ) values were calculated and plotted on the Y-axis against TQ concentrations. The cell culture supernatants were used to measure the secreted protein levels of IL-1β and sPD-L1 via ELISA. The expression and protein levels were compared to the DMSO control. All experiments were performed in triplicate and data were expressed as the mean ± standard deviation. Statistical analysis was performed using the GraphPad Prism software version 9.1.1. The data were analyzed using one-way ANOVA, Dunnett’s multiple comparisons, and unpaired *t*-test.

Within 2–4 h, PBMCs were isolated from fresh blood by the Ficoll density gradient technique using Histopaque (Sigma-Aldrich gradient, St. Louis, MO, USA). Cell viability of PBMCs was investigated using a Trypan Blue dye exclusion test. The PBMCs of each sample were cryopreserved and stored in a freezer at −80 °C for later use (Figure 9). 

### 4.3. PBMCs Pooling 

According to immunohistochemical reports, the current study categorized BC patients into luminal A and TNBC groups. PBMCs of each group of patients were thawed at room temperature, then quickly washed, and centrifuged at 1500 RPM/10 min in cell culture media (RPMI with 10% FBS (Biowest, Nuaillé, France) and 1% Pen-Strep (Gibco by Life Technologies, Grand Island, NY, USA). The supernatant was decanted and pellets were collected (Figure 9).

### 4.4. MDA-MB231 Culture and Tumor-Conditioned Media (TCM) Preparation

MDA-MB 231 (purchased from Vacsera Egypt) was incubated in Dulbecco’s modified Eagle’s medium (DMEM) purchased from Lonza, Cologne, Germany (cat. no. 12-604F), supplemented with 4.5 g/L glucose, 4 mmol/L L-glutamine, 10% FBS, and 1% Pen-Strep at 37 °C with an atmosphere of 5% CO_2_ and 95% humidity. The cultured cells were allowed to become 80% confluent; then, TCM was harvested and centrifuged to remove suspended cells. After that, the supernatant was collected and stored in a freezer for further use. 

### 4.5. Monocyte Isolation from Hormonal BC Patients and Tumor-Associated Macrophage Differentiation In Vitro

Monocytes were isolated from PBMCs using the MojoSort™ Human CD14+ Monocyte Isolation Kit (Biolegend, San Diego, CA, USA, cat. no. 480019, 480048) following the manufacturer’s instructions. The freshly isolated monocytes were plated in a 48-well plate (10,000 cells per well). Monocytes were cultured in TCM and cell culture media with a ratio of 1:1. Additionally, 1 µg/mL of human interleukin-10 (IL-10) (Shenandoah Biotechnology, Warminster, PA, USA, cat. no. 100-83), 1 µg/mL of human interleukin-4 (IL-4) (Shenandoah Biotechnology, Warminster, PA, USA, cat. no. 100-09), and 1 µg/mL of human macrophage colony-stimulating factor (M-CSF) (Shenandoah Biotechnology, Warminster, PA, USA, cat. no. 100-03) were added to the culture media. Monocytes were incubated for seven days. Every other day, medium cytokines were refreshed and cells were microscopically examined; at day 7, TAMs were harvested (3 × 10^4^ cells per well in a 48-well plate) (Figure 9).

### 4.6. Flow Cytometry 

At day 7, flow cytometry was performed using anti-CD163 FITC in accordance with the manufacturer’s instructions (cat. no. sc.33715, Santa Cruz Biotechnology, Dallas, TX, USA) to ensure successful TAMs differentiation. Briefly, cells were dissociated, followed by single-cell suspension preparation (240,000 cells/tube). Cells were washed with 2 mL (PBS 1% FBS) and centrifuged for 5 min; then, the supernatant was discarded. After that, 1.2 µg anti-CD163 FITC was added (5 µg/1 million cells) and incubated for 30 min at 4° degrees. Finally, analysis was performed using CytoFLEX flow cytometry.

### 4.7. Thymoquinone (TQ) Preparations

First, 20, 50, and 100 mM stock solutions of TQ (Acros Organics, Geel, Belgium code:305070010, GC: 98.6%) were dissolved in dimethyl sulfoxide (DMSO) (Loba Chemie, Mumbai, India) and stored at −80 °C. The TQ stock solutions were diluted in fresh cell culture media into (20, 50, and 100 µM) just before use. Note that the percentage of DMSO was 0.1% in order to not be toxic to cells.

### 4.8. PBMCs and TAMs Coculture with TQ

In a pyrogen-free 48-well plate, PBMCs were cocultured with TQ (3 × 10^6^ cells per mL) at increasing concentrations (20, 50, and 100 µM) and durations (24, 48, and 72 h), as shown in Figure 9. TAMs were cocultured (3 × 10^4^ cells per well) with the aforementioned concentrations for 24 h. Notably, each TQ concentration was cultured in triplicate.

### 4.9. RNA Extraction, Complementary DNA (cDNA), and Quantified Real-Time Polymerase Chain Reaction (qRT-PCR)

RNA isolation was performed using the RNeasy Mini Kit (QIAGEN, Hilden, Germany, cat. no. 74104). Total RNA was then converted to cDNA by the Thermo Scientific RevertAid First Strand cDNA Synthesis Kit. The relative expressions of CALR (Hs00189032_m1, cat. no. 4331182), NLRP3 (Hs00918082_m1, cat. no.4331182), PYCARD (Hs01547324_gh, cat. no. 4331182), caspase-1 (Hs00354836_m1, cat no. 4331182), IL-1β (Hs01555410_m1, cat. no. 4331182), and GAPDH (Hs99999905_m1 VIC, cat no. 4326317E) (as a housekeeping gene for normalization) were amplified and quantified on a StepOne Real-Time PCR instrument (Applied Biosystems; Thermo Fisher Scientific Inc., Waltham, MA USA) using TaqMan Real-time PCR (Applied Biosystems; Thermo Fisher Scientific Inc., Waltham, MA USA). For each sample, a reaction mix was prepared according to the manufacturer’s instructions, and 4 µL of the respective cDNA was added. The RT-qPCR run was performed in the standard mode, which consists of two stages: the first stage, where the Taq polymerase enzyme was activated for 10 min at 95 °C, and the second stage, which consisted of 40 amplification cycles. Notably, all PCR reactions, including controls, were run in triplicate reactions. Values were calculated as RQ represented as 2 ^−∆∆CT^ (Figure 9). 

### 4.10. Enzyme-Linked Immunosorbent Assay

The protein release of IL-1β and sPD-L1 in cell culture supernatants was conducted following manufacturer’s instructions using the human IL-1β ELISA kit (cat. no. E-EL-H0149, E-lab science, Houston, TX, USA) and the PD-L1 human ELISA kit (cat. no. K1025-100, Biovision, Milpitas, CA, USA). 

### 4.11. Statistical Analysis

All experiments were performed in triplicate and data are expressed as the mean ± standard deviation. Statistical analysis was performed using the GraphPad Prism software version 9.1.1. The data were analyzed using one-way ANOVA and Dunnett’s multiple comparisons to compare each treated column and the DMSO control column. The column chart was plotted, RQ values were on the Y-axis, and TQ concentrations were drawn on the X-axis. To compare the DMSO control and the two BC subtypes (TNBC and luminal A HR+ BC), the unpaired *t*-test was used (**** *p* < 0.0001; *** *p* < 0.001; ** *p* < 0.01; * *p* < 0.05). 

## 5. Conclusions and Future Insights

The present findings showed that TQ is a novel significant inhibitor of CALR, NLRP3 complex components, as well as IL-1β and sPD-L1 in TAMs and PBMCs of HR+ and TNBC, respectively. The current study sheds light on targeting multiple pro-tumorigenic BC markers using TQ. In addition, it urgently recommends exploring TQ’s clinical impact on HR+ and TNBC patients alone and in combination with atezolizumab. Collectively, TQ might be an excellent multi-strike adjuvant and a potential immunotherapeutic in both HR+ and TNBC patients for future investigation.

## Figures and Tables

**Table 1:** Clinical features of patients with breast cancer.

Patient	Age	Molecular Subtype	Size of Mass, cm	Type	Ki67	Axillary Lymph Node	Treatment
Patient 1	78	Luminal A	3.5 × 3.5 cm	ILC	12%	Negative	Neoadjuvant hormonal therapy Aromatase inhibitor for three months
Patient 2	69	Luminal A	2.5 × 1.8 cm	IDC	3%	Positive	N/A
Patient 3	65	Luminal A	2.5 × 1.6 cm	IDC	15%	Negative	N/A
Patient 4	75	Luminal A	1.5 cm	IDC	7%	Negative	N/A
Patient 5	45	Luminal A	0.8 × 0.4 cm 0.7 × 0.5 cm	IDC	8%	Negative	N/A
Patient 6	53	Luminal A	1.2 × 1 cm	IDC	8%	Positive	N/A
Patient 7	77	Luminal A	1 cm	IDC	5%	Negative	N/A
Patient 8	72	Luminal A	1.2 cm	ILC	12%	Positive	N/A
Patient 9	55	Luminal A	1 × 1.5 cm	IDC	3%	Positive	N/A
Patient 10	30	Luminal A	1.5 cm	IDC	12%	Positive	N/A
Patient 11	60	Luminal A	1 cm	IDC	18%	Positive	Chemotherapy one month before surgery
Patient 12	55	Luminal A	2.8 × 1.6 cm	IDC	12%	Positive	N/A
Patient 13	60	Luminal A	1 cm	IDC	15%	Positive	N/A
Patient 14	60	Luminal A	1.2 cm	IDC	10%	Positive	N/A
Patient 15	65	Luminal A	1 cm	IDC	10%	Positive	N/A
Patient 16	34	Luminal A	1.1 × 1 cm	IDC	12%	Positive	N/A
Patient 17	60	Luminal A	1.5 cm	IDC	7%	Negative	N/A
Patient 18	62	Luminal A	1.3 × 1.5 cm	ILC	15%	Positive	N/A
Patient 19	44	Luminal A	1.2 cm	IDC	14%	Positive	N/A
Patient 20	60	Luminal A	1 cm	IDC	10%	Positive	N/A
Patient 21	64	Luminal A	1.5 cm	ILC	12%	Positive	N/A
Patient 22	44	Luminal A	2.5 × 1.2 cm	IDC	14%	Positive	N/A
Patient 23	62	Luminal A	1.3 cm	IDC	10%	Positive	N/A
Patient 24	47	Luminal A	2 cm	IDC	14%	Positive	N/A
Patient 25	53	Luminal A	1.3 × 1 cm	IDC	12%	Negative	N/A
Patient 26	38	Luminal A	1.5 cm	IDC	7%	Negative	N/A
Patient 27	79	Luminal A	1.5 × 1.2 cm	IDC	8%	Positive	N/A
Patient 28	57	Luminal A	1 cm	IDC	10%	Positive	N/A
Patient 29	59	Luminal A	1.3 cm	IDC	12%	Positive	N/A
Patient 30	66	Luminal A	2 × 1.2 cm	IDC	12%	Positive	N/A
Patient 31	46	TNBC	2 × 1.5 cm 1.1 × 1 cm	IDC	25%	Positive	N/A
Patient 32	51	TNBC	2.5 cm	IDC	60%	Negative	N/A
Patient 33	36	TNBC	1 cm	IDC	50%	Negative	Finished six cycles of chemotherapy
Patient 34	68	TNBC	2.5 × 3.4 cm	IDC	40%	Negative	N/A
Patient 35	73	TNBC	3.5 cm	IDC	60%	Negative	Finished neoadjuvant chemotherapy
Patient 36	60	TNBC	2.5 × 1.5 cm	IDC	50%	Negative	N/A
Patient 37	36	TNBC	4 × 2 × 2 cm	IDC	70%	Negative	N/A
Patient 38	46	TNBC	3.5 cm	IDC	50%	Negative	N/A
Patient 39	60	TNBC	2 × 1.5 cm	IDC	40%	Negative	N/A
Patient 40	72	TNBC	1.2 cm	IDC	50%	Negative	N/A
Patient 41	39	TNBC	1.5 cm	IDC	70%	Positive	N/A
Patient 42	54	TNBC	1 cm	IDC	50%	Negative	N/A
Patient 43	38	TNBC	2 × 1.5 cm	IDC	40%	Negative	N/A
Patient 44	58	TNBC	1.5 cm	IDC	70%	Negative	N/A
Patient 45	72	TNBC	1.2 × 1 cm	IDC	60%	Negative	N/A

Clinical features include the following: age, molecular subtype, tumor size, type, ki67, axillary lymph node status, and treatment. ILC: invasive lobular carcinoma; IDC: invasive ductal carcinoma; N/A: none applicable.

## Data Availability

The data presented in this study are available upon request from the corresponding author.
